# Focus on radioiodine-131 biokinetics: the influence of methylprednisolone on intratherapeutic effective half-life of ^131^I during radioiodine therapy of Graves’ disease

**DOI:** 10.1007/s12020-020-02593-x

**Published:** 2021-01-13

**Authors:** C. Happel, W. T. Kranert, D. Gröner, J. Baumgarten, J. Halstenberg, B. Bockisch, A. Sabet, F. Grünwald

**Affiliations:** 1 Department of Nuclear Medicine, Goethe University Frankfurt, University Hospital, Theodor Stern Kai 7, D-60590 Frankfurt/Main, Germany; 2 Department of Anesthesiology, Alice-Hospital, Darmstadt, Germany

**Keywords:** Radioiodine therapy, Radioiodine uptake test, Thyroid, Effective half-life, Graves’ disease

## Abstract

**Aim:**

Radioiodine therapy (RIT) may trigger the development of Graves’ ophthalmopathy (GO) or exacerbate pre-existing subclinical GO. Therefore, glucocorticoid administration is recommended for patients with pre-existing GO. Aim of this study was to analyze the influence of glucocorticoid therapy with methylprednisolone on intratherapeutic effective half-life (EHL) of radioiodine-131 in patients with Graves’ disease (GD) as recent studies showed an effect for prednisolone.

**Methods:**

In a retrospective study, 264 patients with GD who underwent RIT without any additional antithyroid medication were evaluated. Intrathyroidal EHL was determined pre- and intratherapeutically. Patients with co-existing GO (*n* = 43) received methylprednisolone according to a fixed scheme starting 1 day prior to RIT, patients without GO (*n* = 221) did not receive any protective glucocorticoid medication. The ratios of EHL during RIT and during radioiodine uptake test (RIUT) were compared.

**Results:**

Patients receiving methylprednisolone showed a slight decrease of the mean EHL from 5.63 d (RIUT) to 5.39 d (RIT) (*p* > 0.05). A comparable result was obtained in patients without glucocorticoids (5.71 d (RIUT) to 5.47 d (RIT); *p* > 0.05). The ratios of the EHL between RIT and RIUT failed to show a significant difference between the two groups. EHL is therefore not significantly influenced by an additional protective treatment with methylprednisolone.

**Conclusions:**

In the present study a decreased intrathyroidal EHL under glucocorticoid medication with methylprednisolone could not be detected. Therefore, co-medication with methylprednisolone in patients with GO may be preferred to avoid an intratherapeutic decrease of EHL by accompanying protective glucocorticoides.

## Introduction

Despite a significantly improved alimentary iodine supply in most of the world, the prevalence of benign thyroid diseases is still high [1–4]. Therefore, therapeutic concepts and procedures are under constant development [5–10]. Radioiodine-131 treatment (RIT) was introduced in 1941 by Saul Hertz and has considerably changed the therapeutic management of benign thyroid diseases in favor of a noninvasive therapeutic option [1, 11, 12]. Today, RIT is proven in the treatment of goiter and thyroid nodules [1, 13]. It is associated with fewer adverse events compared to competing procedures, which are surgical intervention and thermal ablative approaches such as radiofrequency, microwave ablation, or high-intensity-focused ultrasound [14–17]. Moreover, concerns over therapeutic use of radioactive pharmaceuticals recently decreased in public perception [18]. RIT is also an integral element of the therapeutic management of Graves’ disease (GD) [1, 19–21]. GD occurs as an autoimmune disease caused by multiple factors including genetic susceptibility, environmental and endogenous factors [22]. The aim of RIT in GD is the elimination of tyreotoxicosis by an ablative therapeutic concept. In 15–33% of the cases, GD is associated with Graves’ ophthalmopathy (GO) as an extrathyroidal manifestation, which additionally contributes to a reduced quality of life [23–25]. GO results from autoimmune inflammation of intraorbital structures and can be induced and intensified by RIT [25–27]. In fact, earlier studies (with insufficient hormone substitution subsequent to RIT) considerably overestimated this effect, and results and interpretation were meanwhile put into perspective by subsequent studies [28]. However, national and international guidelines for RIT recommend protective glucocorticoid therapy for pre-existing GO after the administration of radioiodine-131 [29–32].

The influence of a protective glucocorticoid therapy on intratherapeutic biokinetics of radioiodine-131 in RIT of GD has been controversially discussed for decades, and is still not clearly understood [25, 32–39]. Studies by Halstenberg et al. and Gamstedt et al. concluded that intrathyroidal uptake of radioiodine-131 was significantly reduced by protective glucocorticoid therapy [32, 34]. Hautzel et al. and Berson et al. showed a reduced intratherapeutic intrathyroidal effective half-life (EHL) of radioiodine-131 during RIT of GD with additional protective glucocorticoid medication [37, 38]. In contrast, Jensen et al., Fredrickson et al., and Hill et al. did not find any significant interference of radioiodine-131 biokinetics with administration of glucocorticoids [25, 35, 36].

The aim of the presented study was to evaluate and discuss the influence of protective glucocorticoid medication with methylprednisolone on intratherapeutic intrathyroidal EHL of radioiodine in patients with GD and accompanying GO, since an influence of prednisolone has been previously described [37]. The results were compared to the EHL in the pretherapeutically performed radioiodine-131 uptake test (RIUT) in a retrospective systematic analysis of a comparably large cohort of patient.

## Material and methods

A total of 1075 patients with GD who underwent RIT were evaluated in this systematic retrospective monocentric analysis. The study was approved by the local ethics committee of the University Medical Center Frankfurt/Germany (Nr: 20-777). Inclusion criteria were an on-site RIUT and RIT without antithyroid co-medication or other corticoids over a period of at least 7 days prior to RIUT. Furthermore, patients who did not receive a complete RIUT with at least two measurements of the remaining activity over a period of 1 week were excluded.

The final study cohort consisted of 264 patients (206 f, 58 m) with a median age of 51 years (19–83 years) who underwent RIT for GD. The study cohort was subdivided in two groups for further analysis. Group A consisted of 43 patients with accompanying EO and protective glucocorticoid medication beginning at the time of administration of RIT with a daily dose of 32-mg methylprednisolone for the first 4 days, 16 mg for the following 4 days and a further reduction to 8 mg and 4 mg for 2 weeks each. Group B consisted of 221 patients without GO and therefore without additional glucocorticoid medication (Table 1).

**Table 1:** Comparison of the investigated subgroups

	Disseminated autonomy	GD without glucocorticoids	GD with glucocorticoids	Significance
*n*	134	221	43	–
Mean age [*a*]	63 ± 16	51 ± 16	48 ± 16	*p* > 0.05
Mean EHL in RIUT [d]	6.18 ± 1.1	5.71 ± 1.3	5.63 ± 1.4	*p* > 0.05
Mean EHL in RIT [d]	6.10 ± 1.3	5.47 ± 1.6	5.39 ± 1.5	*p* > 0.05
Median ratio	1.01	0.98	0.97	*p* > 0.05
Range ratio	0.41–1.92	0.32–2.23	0.41–1.58	–

Comparison of the subgroups with and without glucocorticoids

RIUT was performed 1 week prior to RIT in all patients by administration of a radioiodine-131 capsule according to current national guidelines, without the administration of glucocorticoids. After measuring the activity of the capsule right away prior to administration in a dose calibrator and determination of the corresponding count rate with a collimated gamma probe with connected multichannel analyzer (2″ × 2″ NaI(Tl)-detector scintiSPEC; SCINTRONIX), the first measurement of intrathyroidal uptake in the patients was performed 24 or 48 h after administration. The second measurement was performed 72 or 96 h later. Each patient measurement was performed twice for 1 min each to calculate the arithmetic mean. For absolute quantification of the intrathyroidal activity, the same calibrated and collimated gamma probe with connected multichannel analyzer was used. Intrathyroidal EHL of radioiodine-131 was calculated using a monoexponential fit for the time activity curve. A target dose of 250 Gy was used for the first RIT. This target dose was increased to 300 Gy for consecutive treatments in the same patients [29, 30, 40]. Thyroid volume was measured by ultrasound and the required activity was calculated using the Marinelli equation [41].

RIT was performed according to German guidelines [29]. After oral administration of a radioiodine-131 capsule with the previously calculated individual therapeutic activity, daily measurements of the remaining intrathyroidal activity were performed over the complete time of hospitalization. Again an individually calibrated and collimated gamma probe with connected multichannel analyzer was used (2″ × 2″ NaI(Tl)-detector scintiSPEC; SCINTRONIX). The patients were positioned standing in a distance of 6 m to the gamma probe and the torso was shielded with a mobile lead wall of 3 cm to avoid irradiation from outside the thyroid. Intrathyroidal EHL was calculated using a monoexponential fit of the time activity curve.

In order to evaluate methodology of RIUT and RIT as an influencing factor, a further subgroup of 134 patients who received RIUT and RIT for disseminated autonomy of the thyroid in the same period of time was investigated. In this subgroup, RIUT and RIT were performed without protective glucocorticoid medication. The same exclusion criteria as for the subgroups of patients with GD were taken as basis.

For quantitative evaluation the calculated intratherapeutic EHL was correlated with the pretherapeutically calculated EHL, and the resulting ratios were compared. Statistical assessment of the results was performed using BIAS for MS-Windows (^©^epsilon 2014—Version 10). The Wilcoxon–Mann–Whitney test was used to calculate significance for all data. *P* values below 0.05 were considered statistically significant.

## Results

**Fig. 1:**
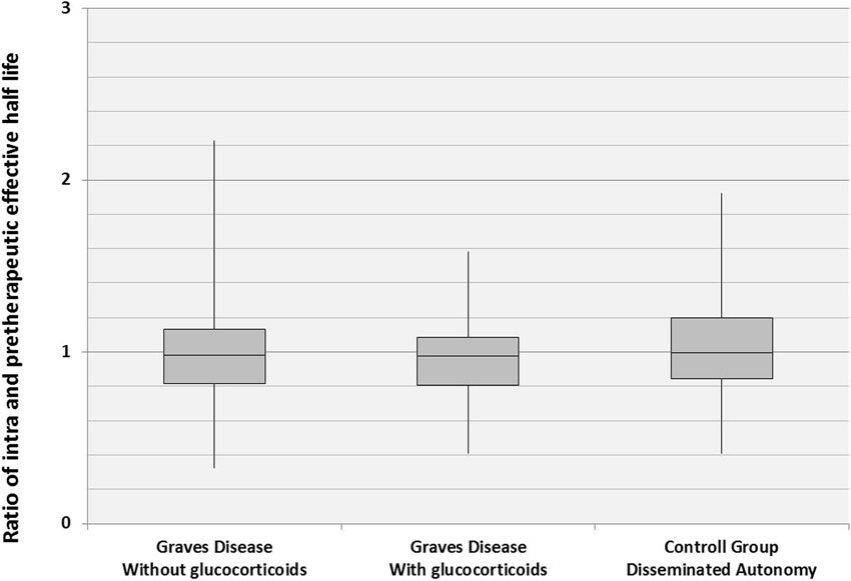
Influence of glucocorticoids on the ratio of intra and pretherapeutic effective half-life in the investigated subgroups

In the group of patients with protective glucocorticoid medication (*n* = 43) the mean EHL was 5.6 ± 1.4 d in RIUT and 5.4 ± 1.5 d during RIT. Although this is a slight numeric decrease of 0.2 d, the statistical analysis did not reach significance (*p* > 0.05) (Table 1 and Fig. 1). The same result was obtained in the group of patients who did not receive protective glucocorticoid medication (*n* = 221). The mean EHL in this group was 5.7 ± 1.3 d in RIUT and 5.5 ± 1.6 d during RIT. Although this was again a slight numeric decrease of 0.2 d, statistical analysis did not show any significant difference between the EHL in RIUT and RIT (*p* > 0.05) (Table 1 and Fig. 1). The statistical analysis also did not show any significant differences between initial pre- and intratherapeutic EHL in the separate groups (*p* > 0.05). Therefore, the EHL was not influenced by administration of methylprednisolone. The evaluation of the additional cohort of 134 patients suffering from disseminated thyroid autonomy did not show a significant difference between intratherapeutic EHL in RIUT and RIT. Mean EHL in this cohort was 6.2 ± 1.1 d in RIUT and 6.1 ± 1.3 d in RIT. The applicability of the used method can therefore be confirmed (Table 1 and Fig. 1).

In order to evaluate potential relative changes in the numeric decrease of the EHL between the two groups, the ratio of intra- and pretherapeutic EHL was calculated for every single patient and for both subgroups in general. These ratios were compared between the two groups. In the group of patients receiving protective glucocorticoid medication, the median ratio of the intratherapeutic EHL related to the pretherapeutic EHL was 0.97 (0.41–1.58) (Table 1 and Fig. 1). Assuming an equal intrathyroidal EHL in RIUT and RIT, this ratio should be 1. In the group of patients without protective glucocorticoid medication, this ratio was 0.98 (0.32–2.23) (Table 1 and Fig. 1). The median ratio in the subgroup of patients suffering from disseminated autonomy was 1.00 (0.41–1.92) (Table 1 and Fig. 1). The statistical comparison of the calculated ratios failed to show a significant difference between the groups (*p* = 0.99).

## Discussion

The results of the presented study are in contradiction to the results of the study of Hautzel et al. [38]. In this study, the authors retrospectively evaluated 315 patients with GD who underwent RIT. Of these 315 patients, 125 suffered from GO and received prednisolone as protective glucocorticoid medication. The other 190 patients did not suffer from GO and did therefore not receive glucocorticoid medication. Intratherapeutic intrathyroidal EHL was compared among the two groups. Hautzel et al. found the EHL of patients receiving prednisolone (5.01 ± 1.44 d) to be significantly reduced compared to the patients without any protective glucocorticoid medication (5.48 ± 1.54 d) (*p* < 0.01) [38]. As potential reason for this significant decrease, Hautzel et al. assumed a glucocorticoid-induced increased renal clearance. Due to this enhanced renal clearance, less plasma radioiodine-131 is available for re-uptake into the thyroid during RIT, directly resulting in a decreased intrathyroidal EHL [38]. The reason for the reduced intrathyroidal EHL may therefore be the diuretic effect of prednisolone. An increased excretion of urine during glucocorticoid medication and therefore a reduced EHL is amply documented and is explained in detail in papers of Fredrickson et al., Berson and Yalow, and Ingbar [36, 37, 42]. Therefore, Hautzel et al. recommended to increase the calculated required activity in RIT by 10–15% in patients with GD and accompanying GO when prednisolone is given as protective glucocorticoid [38].

However, the results of the presented study are not directly comparable to the results of Hautzel et al. [38], as the presented study excluded all patients who did not receive a full RIUT over a period of 4 days. In contrast to that, Hautzel et al. performed RIUT with one single measurement 24 h after administration of the RIUT activity. The authors were therefore not able to calculate an individual pretherapeutic intrathyroidal EHL. A comparison of a potential change of the EHL between RIUT and RIT was therefore not possible [38].

The impact of antithyroid medication is well-described in current literature [43]. Moreover, the compliance of the patients and potential modification of the dosage between RIUT and RIT may have an unknown impact on intrathyroidal biokinetics. Therefore, those patients were excluded from further evaluation in the presented study to avoid compromising results.

The key difference between the presented study and the study of Hautzel et al. is, however, that different glucocorticoids were used. Hautzel et al. administered prednisolone, as recommended e.g., in the guideline of the German Society of Nuclear Medicine [29]. In contrast, methylprednisolone was administered in the presented study. A comparison of both glucocorticoids shows that they do not differ in effective potency (both fivefold compared to cortisol) but differ significantly in their mineralocorticoid potency and therefore in their diuretic effectiveness. Mineralocorticoid potency is 0.6-fold compared to cortisol for prednisolone but absent for methylprednisolone [32, 44]. The contradicting results regarding the intrathyroidal EHL of radioiodine-131 might therefore be explained by the mineralocorticoid potency.

However, despite significantly changed biokinetics, Hautzel et al. and other authors did not find an impact of glucocorticoid medication on the therapeutic outcome of RIT [24–26, 38, 45–47]. In the presented study, the therapeutic outcome was not evaluated. However, a significantly reduced therapeutic effect would be unlikely, as intrathyroidal EHL did not change significantly under protective glucocorticoid medication with methylprednisolone.

A potential selection bias is that only patients suffering from GO received glucocorticoids. Therefore, GO itself cannot be precluded as a potential influencing factor. However, due to ethical reasons this correlation could not be investigated because patients suffering from GO cannot be denied glucocorticoids. The evaluation of an additional cohort of patients suffering from disseminated thyroid autonomy did not show a significant difference between intratherapeutic EHL in RIUT and RIT as expected. The correctness of the used method could therefore be confirmed.

## Conclusion

The results of this study indicate that methylprednisolone does not significantly influence intratherapeutic EHL of radioiodine-131 in the thyroid. Given the fact that a decrease of the EHL has been described for prednisolone [38], methylprednisolone seems to be superior to prednisolone in protective treatment of GO. However, a prospective head-to-head comparison of the two drugs is needed and should be subject of further studies.
